# Diagnostic Value of Lymph Node Biopsy in Adult Patients with Classic Fever of Unknown Origin Accompanied by Lymphadenopathy

**DOI:** 10.3390/jcm15103792

**Published:** 2026-05-14

**Authors:** Huiting Liu, Guiren Ruan, Xiaochun Shi, Xinchao Liu, Ying Ge

**Affiliations:** Department of Infectious Diseases, Peking Union Medical College Hospital, Chinese Academy of Medical Science and Peking Union Medical College, Dongcheng District, Beijing 100730, China; liuhuiting@pumch.cn (H.L.);

**Keywords:** fever of unknown origin, lymphadenopathy, lymph node biopsy, lymphoma

## Abstract

**Background:** Lymph node biopsy is an important means of etiologic diagnosis for patients with fever of unknown origin (FUO) and lymphadenopathy. However, it is an invasive procedure and may yield negative results. It is worth exploring which kinds of patients could benefit most from lymph node biopsy. **Methods**: FUO patients (n = 242) who had lymphadenopathy and underwent lymph node biopsy were enrolled into this retrospective single-center study. Clinical manifestations were documented and risk factors suggestive of an underlying positive lymph node biopsy (providing diagnostic clues) and lymphoma were analyzed. **Results**: The etiologies were as follows: infectious disease in 10 (4.1%) cases, connective tissue diseases in 51 (21.1%) cases, neoplastic diseases in 57 (23.6%) cases, other diseases in 28 (11.6%) cases, and unknown diagnosis in 96 (39.7%) cases. A total of 88 patients (36.4%) were diagnosed through lymph node biopsy. The following four independent risk factors were found to be related to positive lymph node biopsy: male gender (OR 2.471; 95% CI 1.158–5.270; *p* = 0.019), no rash (OR 3.531; 95% CI 1.595–7.816; *p* = 0.002), shape index of the lymph node (OR 8.566; 95% CI 1.035–70.915; *p* = 0.046), and hypoalbuminemia (OR 3.370; 95% CI 1.470–7.728; *p* = 0.004). Later, 146 patients with a confirmed diagnosis (including 57 cases of lymphoma and 89 cases of non-lymphoma) were included in the analysis of lymphoma-related factors. Age older than 45 years (OR 8.663; 95% CI 3.045–24.647; *p* < 0.001), no rash (OR 4.946; 95% CI 1.646–14.859; *p* = 0.004), serositis (OR 3.588; 95% CI 1.137–11.318; *p* = 0.029), abnormal blood flow of the lymph node (OR 3.025; 95% CI 1.034–8.848; *p* = 0.043), abnormal central lymph nodes (OR 6.546; 95% CI 1.721–24.898; *p* = 0.006), focal lesions in the spleen (OR 13.386; 95% CI 2.067–86.706; *p* = 0.006), and serum lactate dehydrogenase (LDH) > 250 U/L (OR 3.885; 95% CI 1.111–13.584; *p* = 0.034) were independent risk factors for lymphoma. **Conclusions**: Lymph node biopsy is a valuable diagnostic procedure for patients with FUO and lymphadenopathy. For male patients without rash, more rounded lymph nodes, and hypoalbuminemia, we strongly recommend lymph node biopsy. Age older than 45 years, no rash, serositis, abnormal blood flow of the lymph node, abnormal central lymph nodes, focal lesions in the spleen, and serum LDH > 250 U/L are risk factors suggesting an underlying lymphoma, and multi-site biopsy should be considered if necessary.

## 1. Introduction

Petersdorf and Beeson proposed the fever of unknown origin (FUO) concept in 1961 [1]. There are well over two hundred potential causes reported in the literature [1,2,3]. FUO may be due to infections, connective tissue diseases (CTD), neoplastic diseases, or miscellaneous disorders. Despite the improvement of diagnostic technology, 20–51% of FUOs elude diagnosis [3,4]. Almost all causes of FUO can be accompanied by lymphadenopathy [5]. Because of non-specific clinical manifestations and radiological features, the etiologic diagnosis of FUO accompanied by lymphadenopathy remains challenging. Timely diagnosis in these patients is important for treatment and prognosis.

Lymphadenopathy is generally considered a core element in the FUO diagnostic process. Lymph node biopsy should be considered for patients remaining undiagnosed despite appropriate investigation [3]. In one study of 69 patients with FUO or inflammation of unknown origin and lymphadenopathy, malignancy accounted for approximately half, and diagnosis was obtained by histological examination of a lymph node biopsy in 80% and by cytology alone in 13% of the patients [6]. However, lymph node biopsy is an invasive procedure with a risk of complications, and sometimes multiple biopsies may be needed to capture diagnostic clues. There is relatively limited research on the diagnostic performance and clinical utility of lymph node biopsy in FUO patients with lymphadenopathy.

Positive lymph node biopsy is essential for a definitive diagnosis. This study endeavors to summarize the characteristics of FUO patients with lymphadenopathy and to explore which kinds of patients could benefit most from lymph node biopsy.

## 2. Methods

Between December 2018 and December 2023, 242 adult patients with classic FUO and lymphadenopathy who met our study criteria underwent a diagnostic lymph node biopsy at Peking Union Medical College Hospital (PUMCH). Classic FUO refers to fevers of 38.3 °C that persist for >3 weeks and remain undiagnosed despite appropriate investigation, with at least 3 outpatient visits or at least 3 days of hospitalization [2]. Patients with nosocomial FUO, immunodeficiency-associated FUO, and travel-associated FUO were excluded. Abnormal lymph nodes were defined as enlargement (greater than 10 mm on short-axis measurements) or hypermetabolism on positron emission tomography–computed tomography (PET/CT), and divided into superficial (cervical and supraclavicular, axillary, and inguinal) and central (mediastinal and retroperitoneal) lymph nodes according to site. Lymph nodes were evaluated by open biopsy or core needle biopsy.

We collected data on patient demographics and clinical, laboratory, imaging, and pathological data. For those who were discharged without definite etiological diagnoses, we carried out at least 6-months of follow-up to clarify the diagnosis. The final etiological diagnosis was independently made by two infectious disease specialists. This study received approval from the institutional ethics committee at PUMCH (No. I-24PJ0373).

Normally distributed variables were represented as means with standard deviation (SD), while not normally distributed variables were indicated by the median and interquartile range (IQR). Categorical variables were reported as percentages (%). For multiple group comparisons, the one-way analysis of variance (ANOVA) test was used for normally distributed data and the Kruskal–Wallis test was used for not normally distributed data, followed by the Bonferroni post hoc test. When comparing categorical data, the Chi-square or Yates’ correction test was utilized. Univariate and multivariable stepwise logistic regression analyses were employed to investigate factors related to positive biopsy and lymphoma. Statistical significance was determined for *p* values < 0.05. Data analysis was carried out using Statistical Package for Social Sciences (SPSS) software version 26.

## 3. Results

### 3.1. Demographic and Clinical Characteristics

A total of 242 patients (161 [66.5%] females and 81 [33.5%] males) were included in this study. The median age at diagnosis was 44 years (range 18–87). The etiologies were as follows: infectious disease in 10 (4.1%) cases, connective tissue diseases in 51 (21.1%) cases, neoplastic diseases in 57 (23.6%) cases, other diseases in 28 (11.6%) cases, and unknown diagnosis in 96 (39.7%) cases. A total of 88 patients (36.4%) were diagnosed through lymph node biopsy (details are listed in Table 1).

The clinical parameters of patients with different etiologies were compared, including demographic characteristics, symptoms and signs, and laboratory and imaging tests (Table 2). Those in the CTD group were more likely to be female and have a rash. The mean age of patients was significantly older in the neoplastic disease group. In the neoplastic disease group, the proportion of patients with focal lesions in the spleen or abnormal central lymph nodes was higher.

### 3.2. Related Factors Associated with Positive Lymph Node Biopsy

Eighty-eight patients with positive lymph node biopsy (providing diagnostic clues) were analyzed further, including 55 cases of lymphoma, 12 cases of necrotizing lymphadenitis, 14 cases of Castleman’s disease, 5 cases of mycobacterium infection, 1 case of IgG4-related disease, and 1 case of sarcoidosis. The univariate analysis findings can be seen in Table 3. Subsequently, variables such as gender, middle-age and older (>45 years old), no rash, serositis, shape index (the ratio between the short and long axes of the node), fusion, poorly demarcated cortex–medulla, abnormal blood flow of the lymph node, abnormal central lymph nodes, splenomegaly/hepatosplenomegaly, focal lesions in the spleen, platelet count, hypoalbuminemia (albumin < 30 g/L), serum lactate dehydrogenase (LDH) > 250 U/L, and renal impairment (eGFR < 90 mL/min/1.73 m^2^) were included in a multivariable logistic regression model. The result identified the following factors as being related to positive biopsy: male (OR 2.471; 95% CI 1.158–5.270; *p* = 0.019), no rash (OR 3.531; 95% CI 1.595–7.816; *p* = 0.002), shape index of the lymph node (OR 8.566; 95% CI 1.035–70.915; *p* = 0.046), and hypoalbuminemia (OR 3.370; 95% CI 1.470–7.728; *p* = 0.004).

### 3.3. Related Factors Associated with Lymphoma

In total, 146 patients with a confirmed diagnosis (including 57 cases of lymphoma and 89 cases of non-lymphoma) were included in the analysis of lymphoma-related factors (Table 4). Aged older than 45 years (OR 8.663; 95% CI 3.045–24.647; *p* < 0.001), without rash (OR 4.946; 95% CI 1.646–14.859; *p* = 0.004), with serositis (OR 3.588; 95% CI 1.137–11.318; *p* = 0.029), abnormal blood flow of the lymph node (OR 3.025; 95% CI 1.034–8.848; *p* = 0.043), abnormal central lymph nodes (OR 6.546; 95% CI 1.721–24.898; *p* = 0.006), focal lesions in the spleen (OR 13.386; 95% CI 2.067–86.706; *p* = 0.006), and serum LDH > 250 U/L (OR 3.885; 95% CI 1.111–13.584; *p* = 0.034) were independent risk factors for lymphoma.

## 4. Discussion

In the present study, we conducted an exploration of the clinical features of FUO patients with lymphadenopathy and which kinds of patients could benefit most from lymph node biopsy. This will provide information on management strategies for FUO patients with lymphadenopathy.

In this study, causes in nearly 40% of the FUO patients with lymphadenopathy remained unknown, but the most common cause was lymphoma (23.6%), similar to in other studies [5,7]. CTD was the second leading cause, which accounted for 21.1% of the population. Early identification of the underlying cause and implementation of appropriate empirical treatment are effective strategies for patients with FUO. Although the clinical manifestations and lymph node features of patients with different etiologies vary, these features were not sufficient to aid diagnosis separately. Some scholars have suggested PET/CT as an approach to differentiate lymphomas [5,8]; however, PET/CT is expensive and is not available at some centers. Currently, lymph node biopsy is still the mainstay of diagnosis for FUO patients with lymphadenopathy. In our in-depth discussion, it is worthwhile to explore which kinds of patients could benefit most from lymph node biopsy through simple clinical parameters.

Our study revealed that male gender and age were the risk factors for positive biopsy and lymphoma, respectively. The association between male gender and positive biopsy results may be attributed to the gender-specific epidemiology of FUO etiologies. In a retrospective analysis including 1641 FUO patients from West China Hospital of Sichuan University, male patients with FUO were more frequently affected by infectious diseases, particularly tuberculosis, as well as hematologic malignancies, which are the primary indications for diagnostic biopsy, while the proportion of female patients with non-infectious inflammatory diseases was much higher than that of male ones [9]. Therefore, the higher rate of positive biopsies in the males in our cohort likely reflects this higher pre-test probability of having structural pathologies amenable to histological diagnosis. As for age, infectious diseases predominated in patients of all ages; the proportion of patients with non-infectious inflammatory diseases was relatively high in patients under 40 years of age; the proportion of neoplastic diseases was relatively high in patients aged 40 and above [9]. In another study, the risk of malignancy was 4% in patients older than 40 and 0.4% in younger patients [10]. This is also consistent with the findings in our study showing that the positive rate of lymph node biopsy is higher among middle-aged and elderly patients.

Regarding the morphological features, we found that rounded lymph nodes correlate with positive biopsy results. A rounded shape of the lymph node, which means a decrease in of the long-to-short axis ratio, is not merely a sonographic feature but a morphological manifestation of architectural disruption. Prativadi et al. summarized the key points of ultrasound differentiation of benign and malignant cervical lymph nodes [11]. Overall, benign reactive nodes tend to preserve an oval or reniform shape, while malignant nodes often become more rounded; the blending of borders of adjacent lymph nodes is suggestive of malignancy; normal and benign nodes will have hilar vascular flow and no peripheral vascularity, while malignant lymph nodes typically have mixed or peripheral vascularity [11]. Although color Doppler ultrasound provides an accurate, sensitive, and specific method for differentiating benign and malignant lymph nodes, a single ultrasound manifestation has limitations in differential diagnosis. Our study included clinical parameters for adjustment and found that lymph nodes with larger shape indexes (more rounded in shape) were more likely to contain diagnostic clues; abnormal blood flow was an independent related factor for lymphoma. Thus, a rounded lymph node in FUO patients serves as a surrogate marker for extensive tissue involvement or malignancy, making it a high-yield target for biopsy.

Although they are not detectable on physical examination, abnormal central lymph nodes are involved in slightly more than half of lymphoma patients [5,12]. In the Zhang L et al. study, the presence of enlarged intra-abdominal lymph nodes was an independent risk factor suggestive of an underlying lymphoma [12]. Some scholars have found that the uptake value of lymphoma groups is significantly higher than that of benign groups, and a high maximum normalized uptake value of the retroperitoneal lymph nodes appears to be significant in explaining the lymphoma prediction model [5]. Similarly, in this study, an abnormal central lymph node was an independent risk factor for lymphoma.

The main causes of focal splenic lesions comprise lymphomas, infarcts, metastases, cysts, ruptures, hemangiomas, abscesses, etc. [13]. Many hematopoietic neoplasms can cause splenic infiltration with splenomegaly and/or focal splenic lesions. In a study of 148 cases who underwent a splenectomy for lesions identified on imaging, the final pathologic diagnosis was malignancy in 93 patients (63%) [14]. Naples R et al. retrospectively reviewed 68 patients who underwent splenectomy for splenomegaly, about 50% of which had an underlying malignancy, with lymphoma being most common [15]. However, diagnostic splenectomy/splenic biopsy is not routine as it has many complications. In our study, focal lesion in the spleen was an independent related factor for lymphoma.

LDH is a cytoplasmic enzyme present in tissues throughout the body. When cells are damaged, they are released into the blood. Due to tissue destruction caused by tumor growth, serum LDH levels have important significance in cancer diagnosis, especially in lymphoma [16]. In one model for predicting malignancy in FUO patients, high LDH was one of the most important parameters [8]. In addition, patients with lymphoma often have concomitant serositis, the incidence of which is about 20~30% [17]. Here, the current study yielded similar results: elevated serum LDH and serositis are risk factors suggesting an underlying lymphoma.

Although lymph node biopsy is a cornerstone in the diagnosis of FUO, the decision of when and in whom to perform this procedure has historically relied heavily on clinical suspicion and the availability of accessible nodes. Our study adds a layer of precision to this decision-making process. Based on the conclusions of our study, the number of futile biopsies in patients with self-limiting or serologically diagnosable conditions might be reduced, thereby optimizing resource utilization and minimizing patient discomfort. We believe this shift from empirical sampling to targeted biopsy represents a meaningful step forward in the management of complex FUO cases.

Our study has some limitations. First, the enrolled patients were hospitalized at Peking Union Medical College Hospital, and the condition of the patients seemed to be more complex and they were more ill; thus, there is the potential for selection bias in this study. Second, the PET/CT could not be further analyzed due to missing data. We admit that PET/CT might provide more evidence for diagnosis prior to lymph node biopsy; however, in this study, it was not carried out for about 24% of the patients, about 16% of the patients underwent this expensive assessment in other hospitals, and some reports have not been standardized. Third, the etiologies of FUO patients with lymphadenopathy are heterogeneous and a rough binary classification of the patients may oversimplify our conclusions. Lastly, this study cannot establish a causal relationship, emphasizing the need for prospective investigations in the future.

## 5. Conclusions

Lymph node biopsy is a valuable diagnostic procedure for patients with FUO and lymphadenopathy. For male patients without rash, more rounded lymph nodes, and hypoalbuminemia, we strongly recommend lymph node biopsy. Age older than 45 years, no rash, serositis, abnormal blood flow of the lymph node, abnormal central lymph nodes, focal lesions in the spleen, and serum LDH > 250 U/L are risk factors suggesting an underlying lymphoma, and multi-site biopsy should be considered if necessary.

## Figures and Tables

**Table 1:** Etiological diagnosis of 242 enrolled patients.

Items	Case Number (%)	
	Total	Diagnosed by Lymph Node Biopsy/Puncture
N	242	88 (36.4%)
Infectious diseases	10 (4.1%)	5 (2.1%)
Lymphoid tuberculosis	3 (1.2%)	2 (0.8%)
Nontuberculous mycobacterial infection	3 (1.2%)	3 (1.2%)
Epstein–Barr virus infection	2 (0.8%)	0
Cytomegalovirus infection	2 (0.8%)	0
Connective tissue diseases	51 (21.1%)	1 (0.4%)
Adult-onset Still’s disease	35 (14.5%)	0
Unclassified connective tissue disease	8 (3.3%)	0
Systemic lupus erythematosus	3 (1.2%)	0
Dermatomyositis	1 (0.4%)	0
Sjogren syndrome	1 (0.4%)	0
IgG4-related disease	1 (0.4%)	1 (0.4%)
Autoinflammatory diseases	1 (0.4%)	0
Vasculitis	1 (0.4%)	0
Neoplastic diseases	57 (23.6%)	55 (22.7%)
Lymphomas	57 (23.6%) †	55 (22.7%)
Other diseases	28 (11.6%)	27 (11.2%)
Castleman’s disease	14 (5.8%)	14 (5.8%)
Necrotizing lymphadenitis	12 (5.0%)	12 (5.0%)
Sarcoidosis	1 (0.4%)	1 (0.4%)
Idiopathic hypereosinophilia	1 (0.4%)	0
Unknown diagnosis	96 (39.7%)	0

† One patient was diagnosed by liver biopsy with negative lymph node pathological finding; one patient was diagnosed by bone marrow biopsy with negative lymph node pathological finding.

**Table 2:** Clinical characteristics of the study patients with different causes.

Items	Infections	Connective Tissue Diseases	Neoplastic Diseases	Others	Unknown Diagnosis	Total
N	10	51	57	28	96	242
Demographic characteristics						
Female, n (%)	6 (60.0%) ^abc^	47 (92.2%) ^b^	29 (50.9%) ^c^	12 (42.9%) ^ac^	67 (69.8%) ^ac^	161 (66.5%)
Age, mean ± SD (y)	48 ± 15 ^ab^	36 ± 14 ^b^	53 ± 16 ^a^	40 ± 15 ^a^	44 ± 15 ^a^	44 ± 16
Symptoms and signs						
Target lymph node						
Poor mobility, n (%)	3 (30.0%) ^a^	9 (17.6%) ^a^	15 (26.3%) ^a^	5 (17.9%) ^a^	12 (12.5%) ^a^	44 (18.2%)
Hard textures, n (%)	1 (10.0%) ^a^	10 (19.6%) ^a^	9 (15.8%) ^a^	3 (10.7%) ^a^	6 (6.3%) ^a^	29 (12.0%)
Tenderness, n (%)	0 (0.0%) ^a^	12 (23.5%) ^a^	5 (8.8%) ^a^	3 (10.7%) ^a^	15 (15.6%) ^a^	35 (14.5%)
Rash, n (%)	3 (30.0%) ^ab^	40 (78.4%) ^c^	14 (24.6%) ^b^	5 (17.9%) ^b^	48 (50.0%) ^a^	110 (45.5%)
Arthritis, n (%)	4 (40.0%) ^a^	16 (31.4%) ^a^	9 (15.8%) ^a^	5 (17.9%) ^a^	18 (18.8%) ^a^	52 (21.5%)
Night sweats, n (%)	2 (20.0%) ^a^	2 (3.9%) ^a^	4 (7.0%) ^a^	1 (3.6%) ^a^	7 (7.3%) ^a^	16 (6.6%)
Weight dropped by more than 5%, n (%)	4 (40.0%) ^a^	11 (21.6%) ^a^	18 (31.6%) ^a^	4 (14.3%) ^a^	24 (25.0%) ^a^	61 (25.2%)
Serositis, n (%)	0 (0.0%) ^ab^	5 (9.8%) ^ab^	16 (28.1%) ^b^	4 (14.3%) ^ab^	9 (9.4%) ^a^	34 (14.0%)
Laboratory and imaging tests						
Abnormal lymph nodes *						
Superficial lymph node, n (%)	10 (100%) ^a^	50 (98%) ^a^	54 (94.7%) ^a^	24 (85.7%) ^a^	90 (93.8%) ^a^	228 (94.2%)
Cervical and supraclavicular, n (%)	7 (70%) ^a^	39 (76.5%) ^a^	49 (86%) ^a^	20 (71.4%) ^a^	64 (66.7%) ^a^	179 (74%)
Axillary, n (%)	6 (60%) ^a^	40 (78.4%) ^a^	40 (70.2%) ^a^	19 (67.9%) ^a^	65 (67.7%) ^a^	170 (70.2%)
Inguinal, n (%)	5 (50%) ^ab^	36 (70.6%) ^b^	31 (54.4%) ^ab^	9 (32.1%) ^a^	51 (53.1%) ^ab^	132 (54.5%)
Central lymph node, n (%)	7 (70%) ^ab^	30 (58.8%) ^a^	52 (91.2%) ^b^	16 (57.1%) ^a^	54 (56.2%) ^a^	159 (65.7%)
Mediastinal, n (%)	7 (70%) ^ab^	22 (43.1%) ^a^	48 (84.2%) ^b^	11 (39.3%) ^a^	32 (33.3%) ^a^	120 (49.6%)
Intra-abdominal, n (%)	5 (50%) ^a^	28 (54.9%) ^a^	50 (87.7%) ^b^	12 (42.9%) ^a^	50 (52.1%) ^a^	145 (59.9%)
Target lymph node						
Long diameters [cm, median (IQR)]	1.70 (1.53, 1.80) ^ab^	2.10 (1.50, 2.90) ^ab^	2.40 (1.80, 3.40) ^a^	2.10 (1.64, 2.55) ^ab^	2.05 (1.50, 2.60) ^b^	2.10 (1.60, 2.80)
Short diameters [cm, median (IQR)]	0.95 (0.63, 1.08) ^ab^	0.70 (0.60, 0.95) ^b^	1.00 (0.70, 1.70) ^a^	0.90 (0.68, 1.20) ^b^	0.75 (0.60, 1.10) ^b^	0.80 (0.60, 1.20)
Shape index † [median (IQR)]	0.57 (0.43, 0.61) ^a^	0.40 (0.28, 0.55) ^a^	0.46 (0.37, 0.59) ^a^	0.44 (0.34, 0.55) ^a^	0.40 (0.29, 0.52) ^a^	0.43 (0.31, 0.56)
Fusion, n (%)	1 (11.1%) ^a^	3 (6.7%) ^a^	11 (23.4%) ^a^	3 (15.0%) ^a^	10 (12.2%) ^a^	28 (13.8%)
Poorly demarcated cortex–medulla, n (%)	1 (10.0%) ^a^	9 (17.6%) ^a^	21 (40.4%) ^a^	7 (29.2%) ^a^	19 (21.3%) ^a^	57 (25.2%)
Cortical thickening, n (%)	5 (50.0%) ^a^	38 (74.5%) ^a^	41 (78.8%) ^a^	20 (83.3%) ^a^	72 (81.8%) ^a^	176 (78.2%)
Abnormal blood flow, n (%)	2 (20.0%) ^a^	22 (43.1%) ^a^	38 (71.7%) ^b^	11 (45.8%) ^ab^	41 (46.1%) ^a^	114 (50.2%)
Splenomegaly/hepatosplenomegaly, n (%)	5 (50.0%) ^a^	28 (54.9%) ^a^	35 (61.4%) ^a^	9 (32.1%) ^a^	40 (41.7%) ^a^	117 (48.3%)
Focal lesions in the spleen, n (%)	1 (10%) ^ab^	1 (2.0%) ^a^	15 (26.3%) ^b^	0 (0%) ^a^	6 (6.3%) ^a^	23 (9.5%)
White blood cell count [×10^9^/L, median (IQR)]	9.16 (4.79, 11.13) ^a^	8.18 (6.05, 12.86) ^a^	6.03 (3.62, 9.91) ^a^	5.77 (2.80, 8.48) ^a^	7.00 (5.23, 10.04) ^a^	6.75 (4.66, 10.63)
Neutrophil count [×10^9^/L, median (IQR)]	5.73 (2.31, 7.61) ^ab^	6.30 (4.29, 10.62) ^a^	4.07 (2.29, 7.91) ^ab^	3.74 (1.72, 6.26) ^b^	4.71 (3.18, 8.03) ^ab^	4.81 (2.81, 8.01)
Lymphocyte count [×10^9^/L, median (IQR)]	1.69 (0.91, 2.88) ^a^	1.06 (0.76, 1.45) ^b^	0.92 (0.49, 1.38) ^b^	0.99 (0.82, 1.45) ^ab^	1.27 (0.85, 1.65) ^ab^	1.09 (0.73, 1.57)
Hemoglobin [×10^9^/L, median (IQR)]	84.5 (67.5, 103) ^a^	92 (83, 107) ^ab^	93 (78, 106) ^a^	95.5 (86.5, 120.5) ^b^	99 (85, 114.75) ^ab^	94 (81, 112)
Platelet count [×10^9^/L, median (IQR)]	201 (106.5, 350.75) ^ab^	227 (191.5, 364) ^a^	161 (71, 268) ^b^	212 (131.5, 327) ^ab^	244.5 (187.75, 334.75) ^ab^	222 (149.75, 327.25)
Total bilirubin [μmol/L, median (IQR)]	11.7 (6.75, 14.1) ^ab^	7.4 (6, 9.6) ^ab^	11.6 (7.6, 18.5) ^a^	8.75 (7.05, 11.625) ^ab^	7.75 (6.675, 10.1) ^b^	8.30 (6.43, 11.68)
Direct bilirubin [μmol/L, median (IQR)]	3.8 (2.175, 5.8) ^ab^	2.8 (2, 3.5) ^a^	4.4 (3.2, 9.9) ^ab^	3.4 (2.15, 4.925) ^ab^	2.95 (2.4, 3.725) ^b^	3.20 (2.33, 4.68)
Alanine aminotransferase [U/L, median (IQR)]	25 (12.5, 47.25) ^a^	25 (16, 58.5) ^a^	28 (16, 39) ^a^	23 (15.75, 51.5) ^a^	18 (12, 30) ^a^	23 (13, 41)
Aspartate aminotransferase [U/L, median (IQR)]	29 (14, 54) ^ab^	47 (32, 109.5) ^a^	31 (20, 57) ^ab^	30.5 (24.75, 46) ^ab^	31 (19, 57) ^b^	33 (22.25, 64)
Serum albumin < 30 g/L, n (%)	3 (30.0%) ^ab^	11 (21.6%) ^ab^	26 (45.6%) ^b^	6 (21.4%) ^ab^	15 (15.6%) ^a^	61 (25.2%)
Serum lactate dehydrogenase > 250 U/L, n (%)	5 (50.0%) ^ab^	49 (96.1%) ^c^	46 (80.7%) ^bc^	12 (42.9%) ^a^	59 (61.5%) ^ab^	171 (70.7%)
eGFR ‡ < 90 mL/min/1.73 m^2^, n (%)	4 (40.0%) ^ab^	4 (7.8%) ^a^	21 (36.8%) ^b^	4 (14.3%) ^ab^	19 (19.8%) ^ab^	52 (21.5%)
High-sensitivity C-reactive protein > 8 mg/L, n (%)	10 (100.0%) ^a^	42 (82.4%) ^a^	50 (87.7%) ^a^	20 (71.4%) ^a^	83 (86.5%) ^a^	205 (84.7%)

Significance is denoted by alphabetical letterings; groups with significance do not share the same letters. eGFR, estimated glomerular filtration rate. ‡ With the chronic kidney disease epidemiology collaboration. † Shape index, the ratio between the short and long axes of the node. * Abnormal lymph nodes, enlargement (greater than 10 mm on short-axis measurements) or hypermetabolism on PET/CT.

**Table 3:** Factors associated with positive lymph node biopsy findings in patients with FUO.

Variable	Univariate Analysis		Multivariable Analysis	
	Odds Ratio # (95% CI)	*p* Value	Odds Ratio # (95% CI)	*p* Value
Demographic characteristics				
Male	4.046 (2.297–7.126)	<0.001	2.471 (1.158–5.270)	0.019
Aged older than 45 years	2.507 (1.466–4.290)	0.001		
Symptoms and signs				
Target lymph node				
Poor mobility	1.593 (0.822–3.088)	0.168		
Hard textures	1.495 (0.683–3.274)	0.315		
Tenderness	0.470 (0.204–1.086)	0.077		
Without rash	5.246 (2.876–9.566)	<0.001	3.531 (1.595–7.816)	0.002
Arthritis	0.728 (0.377–1.406)	0.345		
Night sweats	1.825 (0.660–5.047)	0.246		
Weight dropped by more than 5%	1.186 (0.653–2.155)	0.576		
Serositis	2.218 (1.066–4.613)	0.033		
Laboratory and imaging tests				
The target lymph node				
Shape index †	7.051 (1.723–28.860)	**0.007**	8.566 (1.035–70.915)	0.046
Fusion	2.585 (1.151–5.806)	0.021		
Poorly demarcated cortex–medulla	2.234 (1.208–4.130)	0.010	2.195 (0.981–4.908)	0.056
Cortical thickening	0.817 (0.425–1.570)	0.544		
Abnormal blood flow	1.841 (1.059–3.200)	0.031	1.956 (0.953–4.012)	0.067
Abnormal central lymph nodes ‡	2.354 (1.301–4.259)	**0.005**		
Splenomegaly/hepatosplenomegaly	1.033 (0.612–1.745)	0.903		
Focal lesions in the spleen	3.750 (1.520–9.250)	**0.004**	2.990 (0.925–9.668)	0.067
White blood cell count	0.965 (0.921–1.011)	0.138		
Hemoglobin	0.998 (0.985–1.011)	0.777		
Platelet count	0.998 (0.996–1.000)	0.039		
Alanine aminotransferase	0.999 (0.994–1.003)	0.557		
Serum albumin < 30 g/L	2.700 (1.489–4.895)	0.001	3.370 (1.470–7.728)	0.004
Serum lactate dehydrogenase > 250 U/L	0.565 (0.318–1.007)	0.053		
eGFR * < 90 mL/min/1.73 m^2^	2.284 (1.224–4.260)	0.009		
High-sensitivity C-reactive protein > 8 mg/L	0.869 (0.419–1.801)	0.706		

† Shape index, the ratio between the short and long axes of the node. ‡ Abnormal lymph nodes, enlargement (greater than 10 mm on short-axis measurements) or hypermetabolism on PET/CT of central (mediastinal and retroperitoneal) lymph nodes. * eGFR, estimated glomerular filtration rate. # Odds Ratio, represents the comparison between patients with positive lymph node biopsy and those with negative biopsy results.

**Table 4:** Factors associated with a diagnosis of lymphoma in patients with FUO.

Variable	Univariate Analysis		Multivariable Analysis	
	Odds Ratio # (95% CI)	*p* Value	Odds Ratio # (95% CI)	*p* Value
Demographic characteristics				
Male	2.615 (1.300–5.261)	0.007		
Aged older than 45 years	5.868 (2.831–12.161)	<0.001	8.663 (3.045–24.647)	<0.001
Symptoms and signs				
Target lymph node				
Poor mobility	1.513 (0.685–3.339)	0.306		
Hard textures	1.004 (0.403–2.501)	0.992		
Tenderness	0.474 (0.162–1.386)	0.173		
Without rash	3.596 (1.727–7.485)	0.001	4.946 (1.646–14.859)	0.004
Arthritis	0.480 (0.205–1.122)	0.090		
Night sweats	1.268 (0.326–4.935)	0.732		
Weight dropped by more than 5%	1.700 (0.800–3.615)	0.168		
Serositis	3.469 (1.411–8.525)	0.007	3.588 (1.137–11.318)	0.029
Laboratory and imaging tests				
Target lymph node				
Shape index †	4.594 (0.790–26.726)	**0.090**		
Fusion	2.801 (1.016–7.723)	0.046	4.157 (0.759–22.761)	0.101
Poorly demarcated cortex–medulla	2.710 (1.258–5.839)	0.011		
Cortical thickening	1.302 (0.571–2.966)	0.531		
Abnormal blood flow	3.619 (1.731–7.566)	0.001	3.025 (1.034–8.848)	0.043
Abnormal central lymph nodes ‡	7.064 (2.571–19.407)	<0.001	6.546 (1.721–24.898)	0.006
Splenomegaly/hepatosplenomegaly	1.780 (0.905–3.502)	0.095		
Focal lesions in the spleen	15.536 (3.396–71.081)	<0.001	13.386 (2.067–86.706)	0.006
White blood cell count	0.990 (0.938–1.045)	0.715		
Hemoglobin	0.990 (0.973–1.006)	0.214		
Platelet count	0.997 (0.994–0.999)	0.012		
Alanine aminotransferase	0.994 (0.987–1.001)	0.120		
Serum albumin < 30 g/L	2.894 (1.407–5.949)	**0.004**		
Serum lactate dehydrogenase > 250 U/L	1.394 (0.617–3.152)	0.425	3.885 (1.111–13.584)	0.034
eGFR * < 90 mL/min/1.73 m^2^	3.743 (1.661–8.432)	0.001		
High-sensitivity C-reactive protein > 8 mg/L	1.968 (0.725–5.339)	0.184		

† Shape index, the ratio between the short and long axes of the node. ‡ Abnormal lymph nodes, enlargement (greater than 10 mm on short-axis measurements) or hypermetabolism on PET/CT of central (mediastinal and retroperitoneal) lymph nodes. * eGFR, estimated glomerular filtration rate. # Odds Ratio, represents comparison of patients with a final diagnosis of lymphoma to those with non-lymphoma diagnoses.

## Data Availability

The datasets analyzed during the current study are not publicly available because the database is still under ongoing analysis, but is available from the corresponding author upon reasonable request.

## Abbreviations

FUO, fever of unknown origin; CTD: connective tissue disease; PET/CT, positron emission tomography–computed tomography; PUMCH, Peking Union Medical College Hospital; SD, standard deviation; IQR, interquartile range; ANOVA, one-way analysis of variance; LDH, lactate dehydrogenase.
